# Supplementary material for: Preventing light-induced toxicity in a new mouse model of sector retinitis pigmentosa caused by Rhodopsin M39R variant
Source: Cell Death Discov. 2025 Oct 21;11:477. doi: 10.1038/s41420-025-02769-2 (PMC12540888; doi:10.1038/s41420-025-02769-2)
Supplement: Supplementary file 1 — Tables and supplementary figures [file 41420_2025_2769_MOESM1_ESM.docx]

**Supplemental Table 1**

| **gRNA** | GAACATGTACGCTGCCAGCATGG |
| --- | --- |
| **donor template** | GTGGTGCGGAGCCCCTTCGAGCAGCCGCAGTACTACCTGGCG  GAACCATGGCAGTTCTC**t**A**g**GCTGGCAGCGTACATGTTCCTGCT  CATCGTGCTGGGCTTCCCCATCAACTTCCTCACGCTC |

ATG (Met) -> A**g**G (Arg)

Change NGG to NAG (PAM site) TCC ->TC**t**

**Supplemental Table 2**

| **Protein effect** | **Nucleotide change** | **Location** | **Sanger Sequencing** |
| --- | --- | --- | --- |
| Met39Arg | c.116T>G | TM helix I | **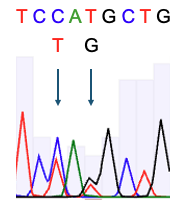** |

**Supplemental Table 3**

**
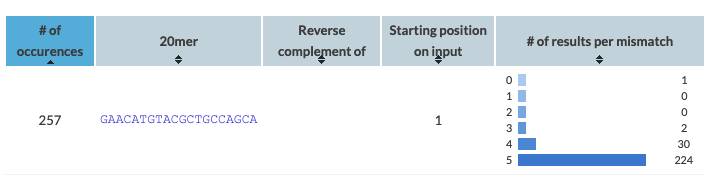
**

In silico prediction of gRNA Off-target occurrences using Off-Spotter (<https://cm.jefferson.edu/Off-Spotter/>). *Rho* was the only gene listed with 0 mismatch.

**Supplemental Table 4**

| **Antibody** | **Host** | **Supplier** | **Working concentration** |
| --- | --- | --- | --- |
| anti-rhodopsin, Rho-4D2 | Mouse | Millipore, MABN15 | WB 1:1000  IHC 1:1000 |
| anti-rhodopsin, Rho-1D4 | Mouse | Professor Molday, UBC | 1:1000 |
| Anti-Iba1 | Goat | Abcam, ab5076 | 1:100 |
| Anti-GFAP | Rabbit | Dako, GA524 | 1:500 |
| Alex Fluor 488 or 555 | Donkey anti-mouse or rabbit | Thermo Fisher Scientific (A32790 or A31572) | 1:1000 |

**
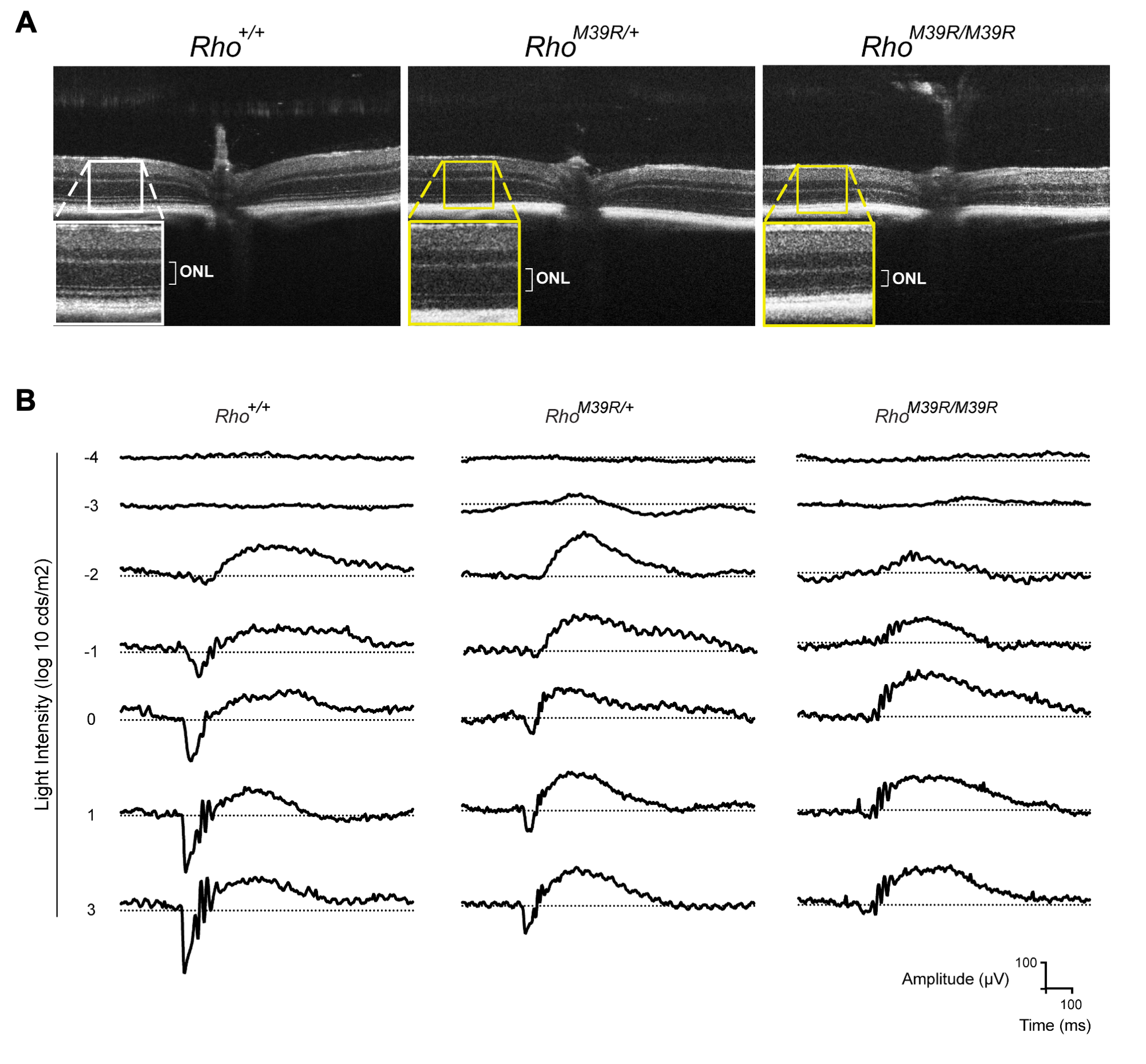
**

**Supplementary Figure 1.** (**A**) Representative images of 3 weeks old *Rho^+/+^*, *Rho^M39R/+^* KI and *Rho^M39R/M39R^* KI mouse retina acquired by OCT using Bioptigen SD-OCT. The inset shows the ONL at higher magnification. ONL = Outer Nuclear Layer. (**B**) ERG recorded at different light intensities using a Celeris ERG system (Diagnosys LLC).

**
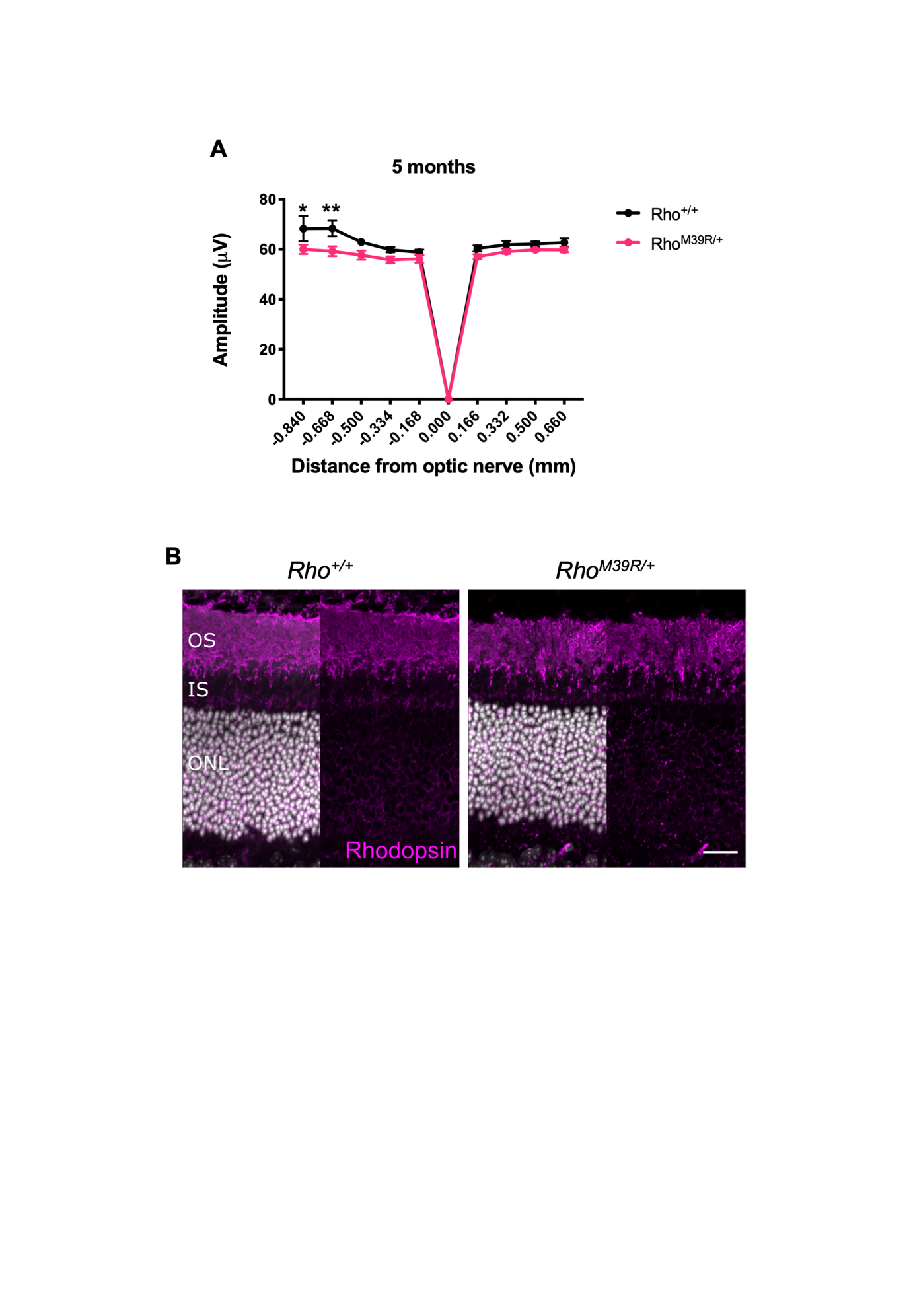
**

**Supplementary Figure 2.** (**A**) ONL thickness was measured by OCT in the central retina of *Rho^+/+^* and *Rho^M39R/+^* KI at 5 months. Mean ± SEM. Two-way ANOVA. Sidak’s multiple comparisons test (* p<0.05, ** p<0.01). *Rho^+/+^* N=4, *Rho^M39R/+^* N=3. (**B**) Rodent eyes were fixed in 4% PFA, incubated in 30% sucrose for 1-2 days, embedded in OCT (embedding matrix), cryosectioned and stained with DAPI and anti-rhodopsin-1D4 antibody. Scale bar=20μm.


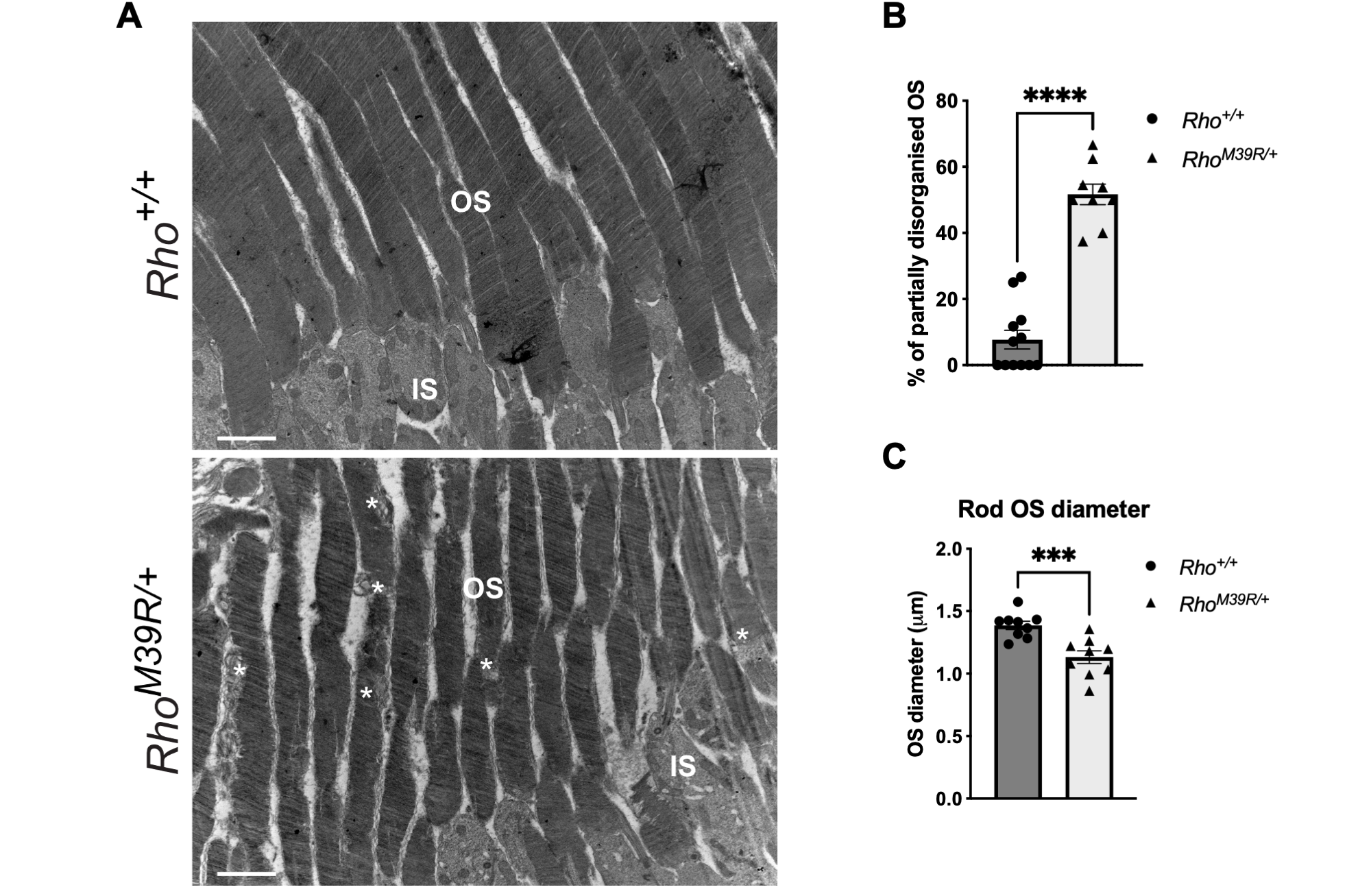


**Supplementary Figure 3.**  (**A**) Representative images of *Rho^+/+^* and *Rho^M39R/+^* KI mouse retina acquired by TEM. Stars indicate disorganised areas along the RO structure. OS = Outer Segment, IS = Inner Segment. Scale bar=2μm. (**B**) The percentage of partially disorganised RO in *Rho^+/+^* and *Rho^M39R/+^* KI mouse retina was measured in TEM pictures and plotted. Mean ± SEM. Mann-Whitney test. 9 pictures from 2 independent animals. (**C**) The diameter of the OS was measured in *Rho^+/+^* and *Rho^M39R/+^* KI mouse retina. Mean ± SEM. Mann-Whitney test. 12-9 pictures from 2 independent animals.

**Supplementary Figure 4** (**A**) Immunoblot of 3 weeks old *Rho^+/+^*, *Rho^M39R/+^* and *Rho^M39R/M39R^* KI mouse retina stained with rhodopsin-4D2 antibody. Anti-GAPDH was used as a loading control. (**B**) Plot of rhodopsin-4D2 relative intensity (34kDa band) measured using Fiji. Values were normalised on GAPDH intensity signal. Mean ± SD. One-way ANOVA. Tukey’s multiple comparisons test between groups. N=3 (* p<0.05).


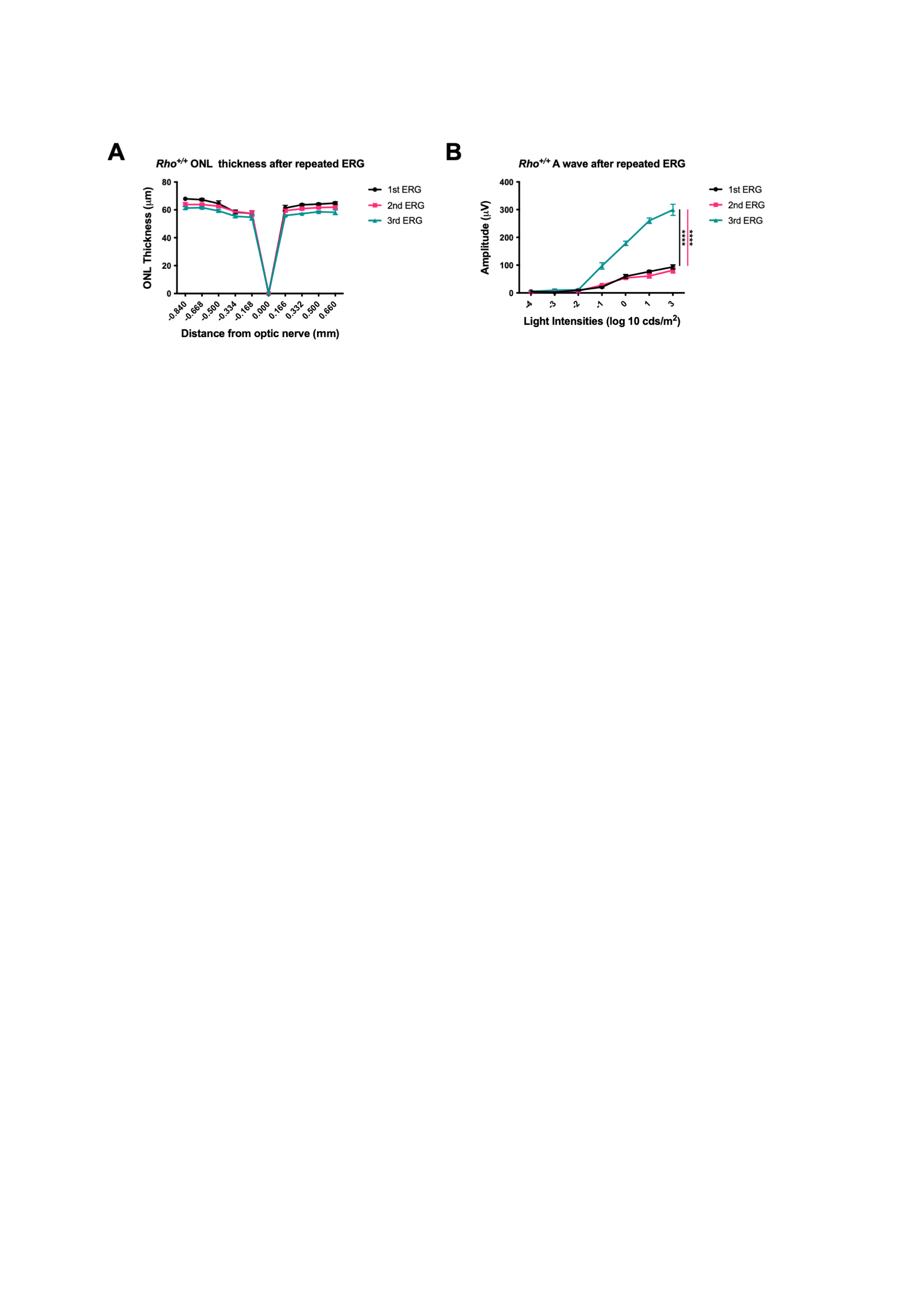


**Supplementary Figure 5.** (**A**) ONL thickness of *Rho^+/+^* after 3 different rounds of ERG. The values were measured by OCT. Mean ± SEM. Mixed-effect ANOVA. Tukey’s multiple comparisons test between groups N=4 (**B**) The scotopic A was also measured in *Rho^+/+^*. Mean ± SEM. Mixed-effect ANOVA. Tukey’s multiple comparisons test between groups. N=4.


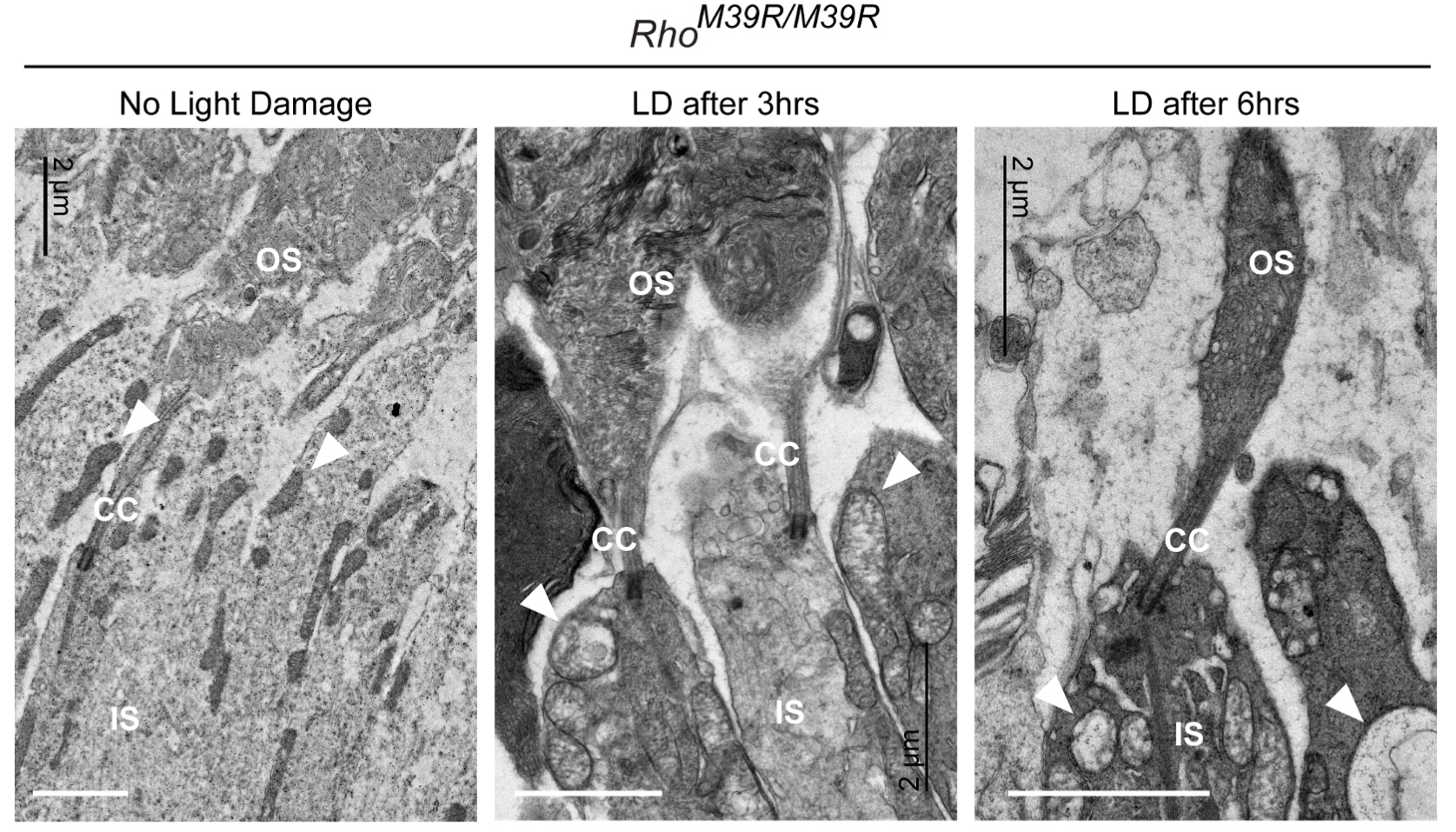


**Supplementary Figure 6.** TEM of *Rho^M39R/M39R^* KI mouse showing the ultrastructure of both OS and IS. Arrows indicate mitochondria. OS = Outer Segment, CC = connecting cilium, IS = Inner Segment. Scale bar=2μm.

**
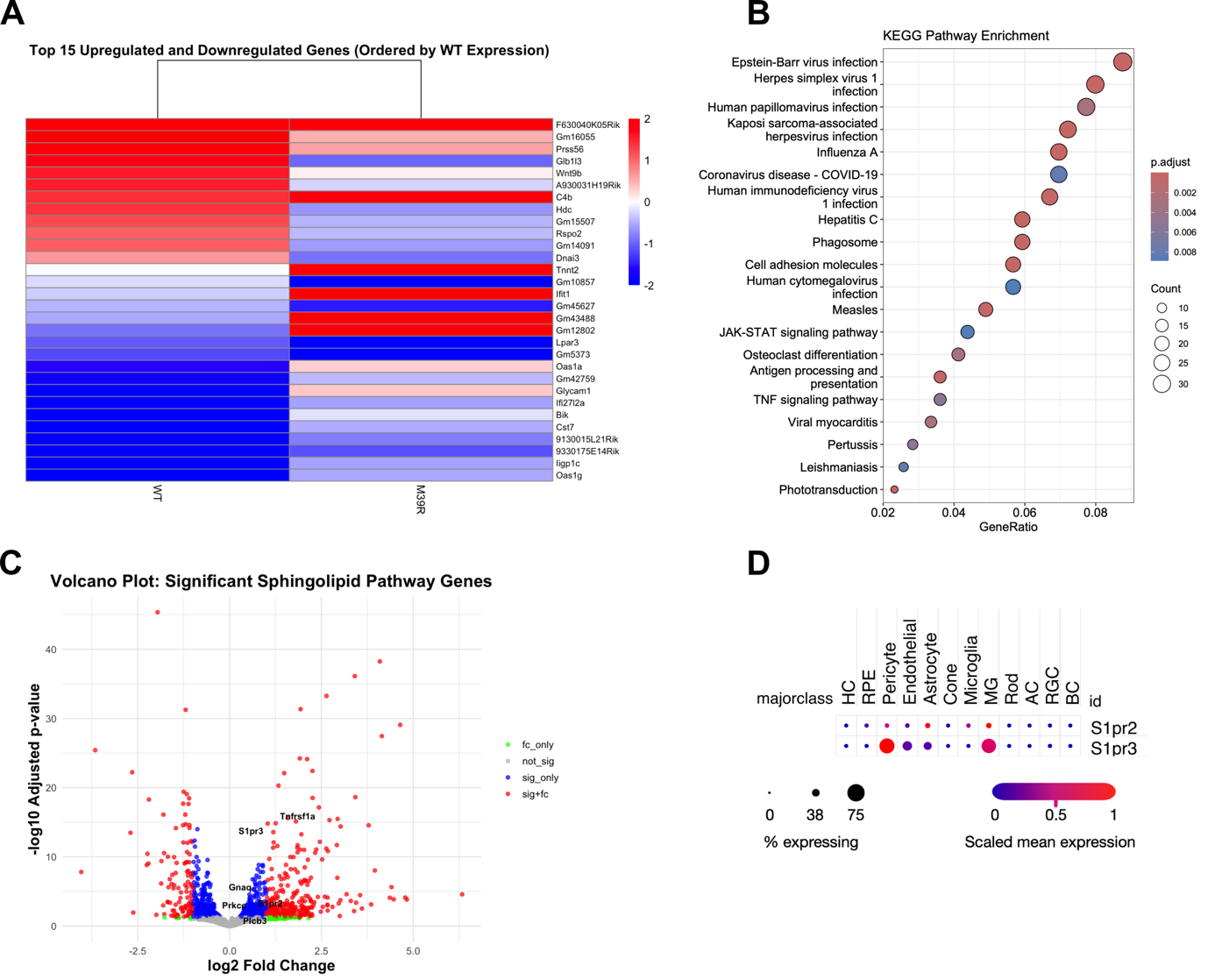
Supplementary Figure 7.** Transcriptomic analysis of *Rho^M39R/M39R^* KI mouse compared to *Rho****^+/+^*.** (**A**) Heatmap of the top 15 upregulated and downregulated genes in *Rho^M39R/M39R^* KI mouse compared to *Rho^+/+^*. (**B**) KEGG enrichment pathway analysis. (**C**) Volcano plot of sphingolipids signalling pathway genes upregulated in our cohort. fc_only = only fold change; not_sign = not significant; sig_only = only significant; sig+fc = significant plus fold change. (**D**) *S1pr2* and *S1pr3* transcripts levels from Single cell RNA sequencing data in wild-type mice extracted from the Broad Single Cell Portal (SCP) <https://singlecell.broadinstitute.org/single_cell/study/SCP2560>. The size and colour of the dots shows the % of cells expressing the transcript and scaled mean expression. HC = horizontal cells; RPE= retinal pigment epithelium; MG = Muller glia; AC = amacrine cells; RGC = retinal ganglion cells; BC = bipolar cells.

**
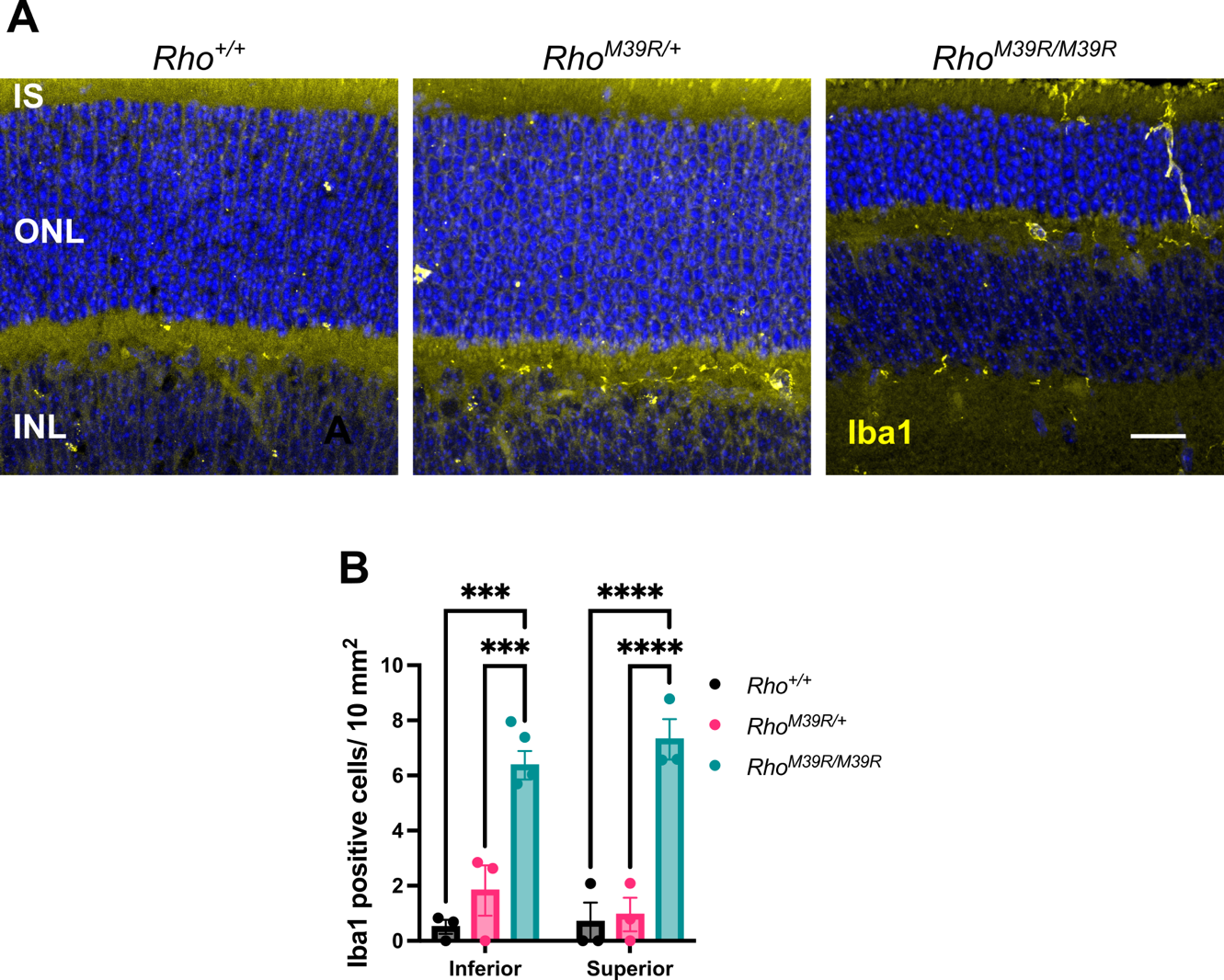
**

**Supplementary Figure 8.** (**A**) IHC of *Rho^+/+^, Rho^M39R/+^*, *Rho^M39R/M39R^* inferior retina in ambient light. The cryosections were stained with DAPI (blue) and anti-Iba1 (yellow). Scale bar=20μm. (**B**) The number of Iba1-positive cells in the ONL were counted. Mean ± SEM. Two-way ANOVA. Tukey’s multiple comparisons test. (**** p<0.0001, *** p<0.001). N=3.

**
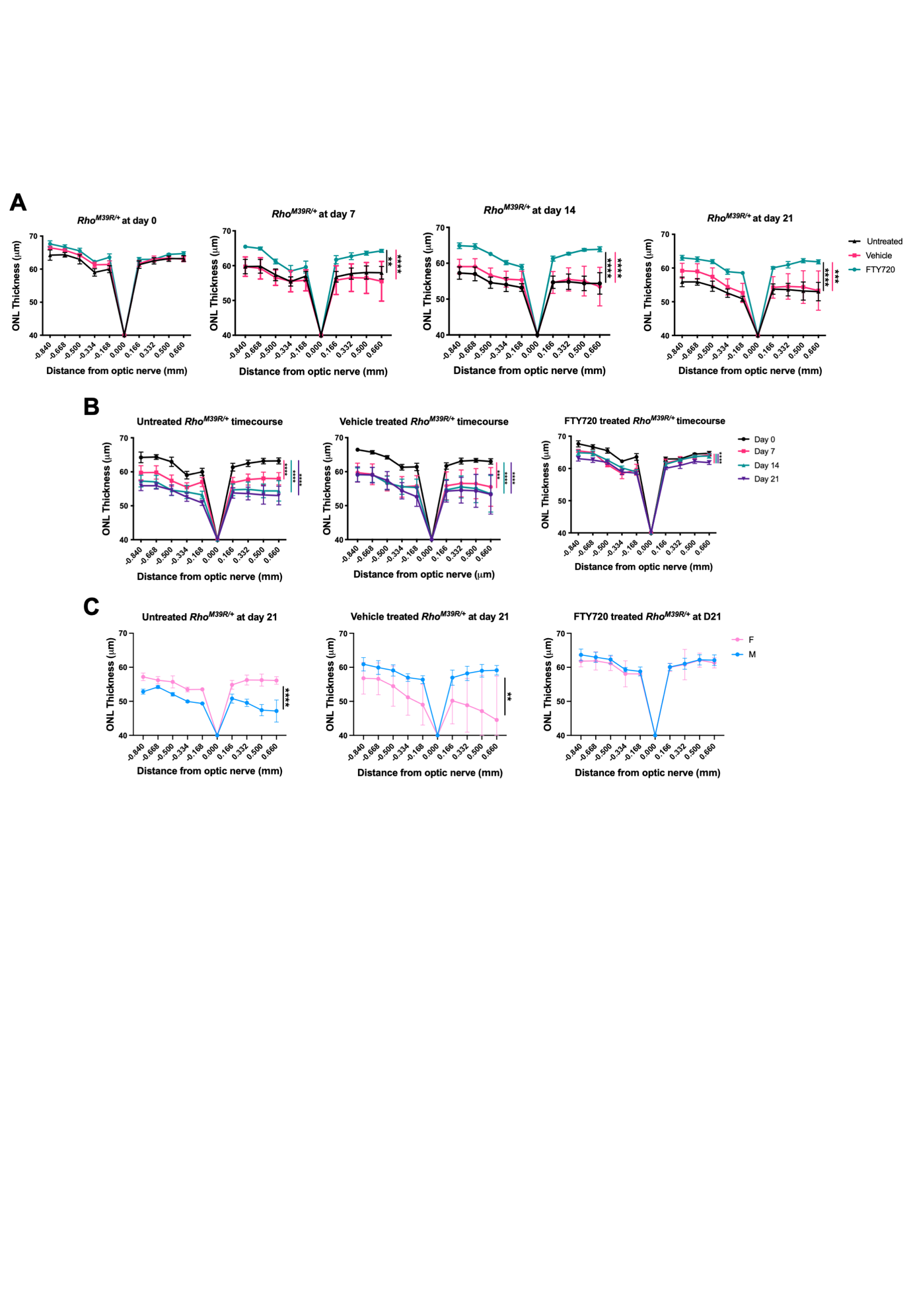
Supplementary Figure 9.** (**A**) ONL thickness of *Rho^M39R/+^* KI mouse retina at each round of ERG: day 0, day 7, day 14 and day 21. Dark-adapted mice were intraperitoneally injected with 10 mg/Kg of FTY20 or with vehicle (saline solution) 30 min prior to the ERG. Untreated mice were also analysed. Mean ± SEM. Two-way ANOVA. Tukey’s multiple comparisons test between groups. (**** p<0.0001, *** p<0.001, ** p<0.01). (**B**) ONL thickness of untreated, vehicle treated and FTY20 treated *Rho^M39R/+^* KI mice measured over time. Mean ± SEM. Mixed-effect analysis. Dunnett’s multiple comparisons test between groups. (**** p<0.0001, *** p<0.001). Untreated N=5, vehicle treated N=7, FTY720 treated N=6.


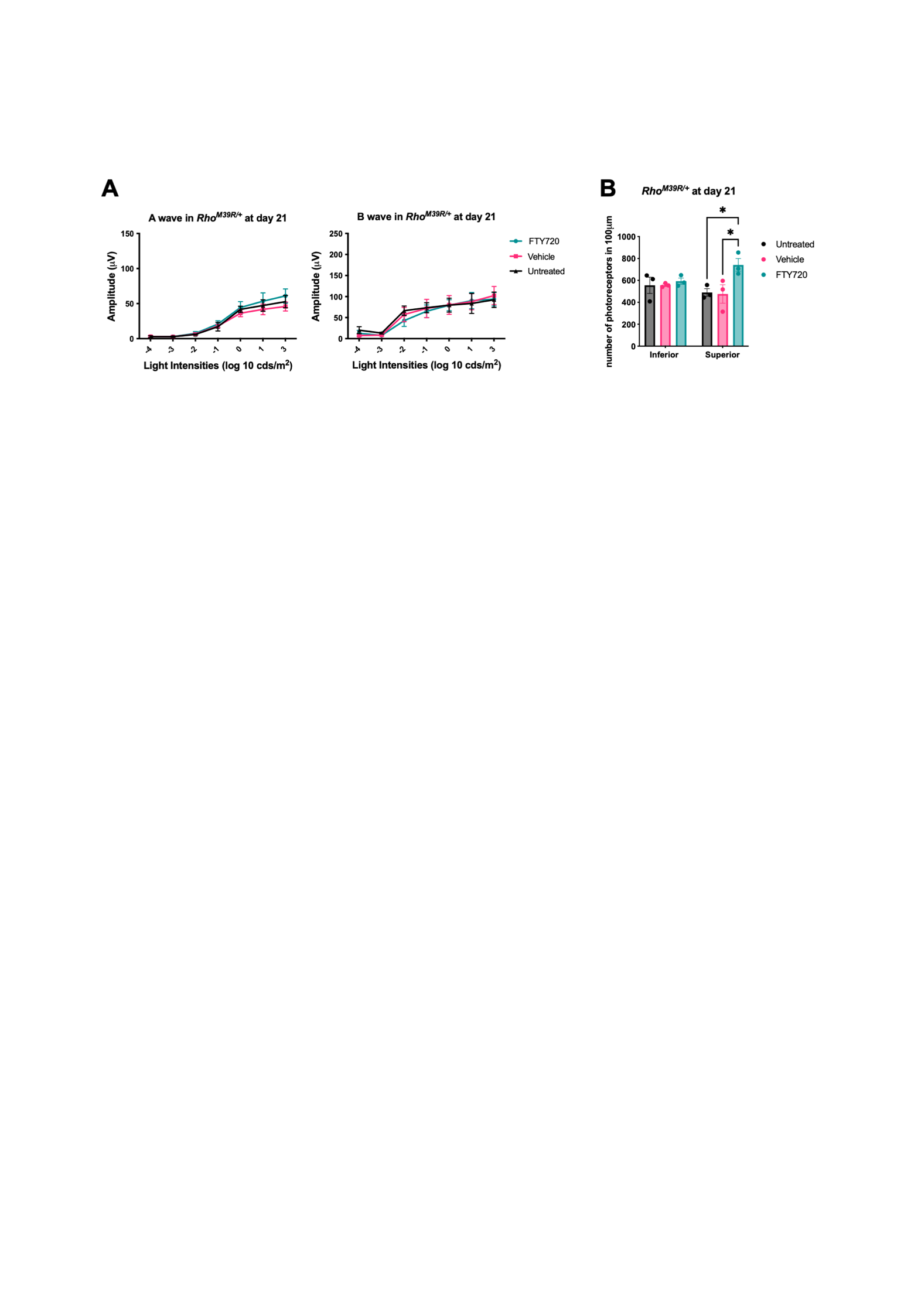


**Supplementary Figure 10.** **(A)** A and B wave of untreated, vehicle or FTY70 treated *Rho^M39R/+^* KI mice measured at the end of the light damage assay (day 21). Mean ± SEM. Two-way ANOVA. Tukey’s multiple comparisons test between groups. N=3/4. (**B**) IHC of untreated, vehicle or FTY70 treated *Rho^M39R/M39R^* superior retina after light damage. The cryosections were stained with DAPI. The number of photoreceptors in the ONL was measured at 200 to 400 μm from the optic nerve in the inferior and superior retina. The analysis was performed on images of the central retina acquired with a microscope EVOS FL auto 2. The area of 10-20 nuclei per retina was measured and divided to total area of the ONL to calculate the total number of nuclei. The number of photoreceptors in 100 μm per treated/untreated animal was plotted. Mean ± SEM. Two-way ANOVA (* p<0.05, ** p<0.01). N=3. Two-way ANOVA. Tukey’s multiple comparisons test between groups. (* p<0.05). N=3.

**
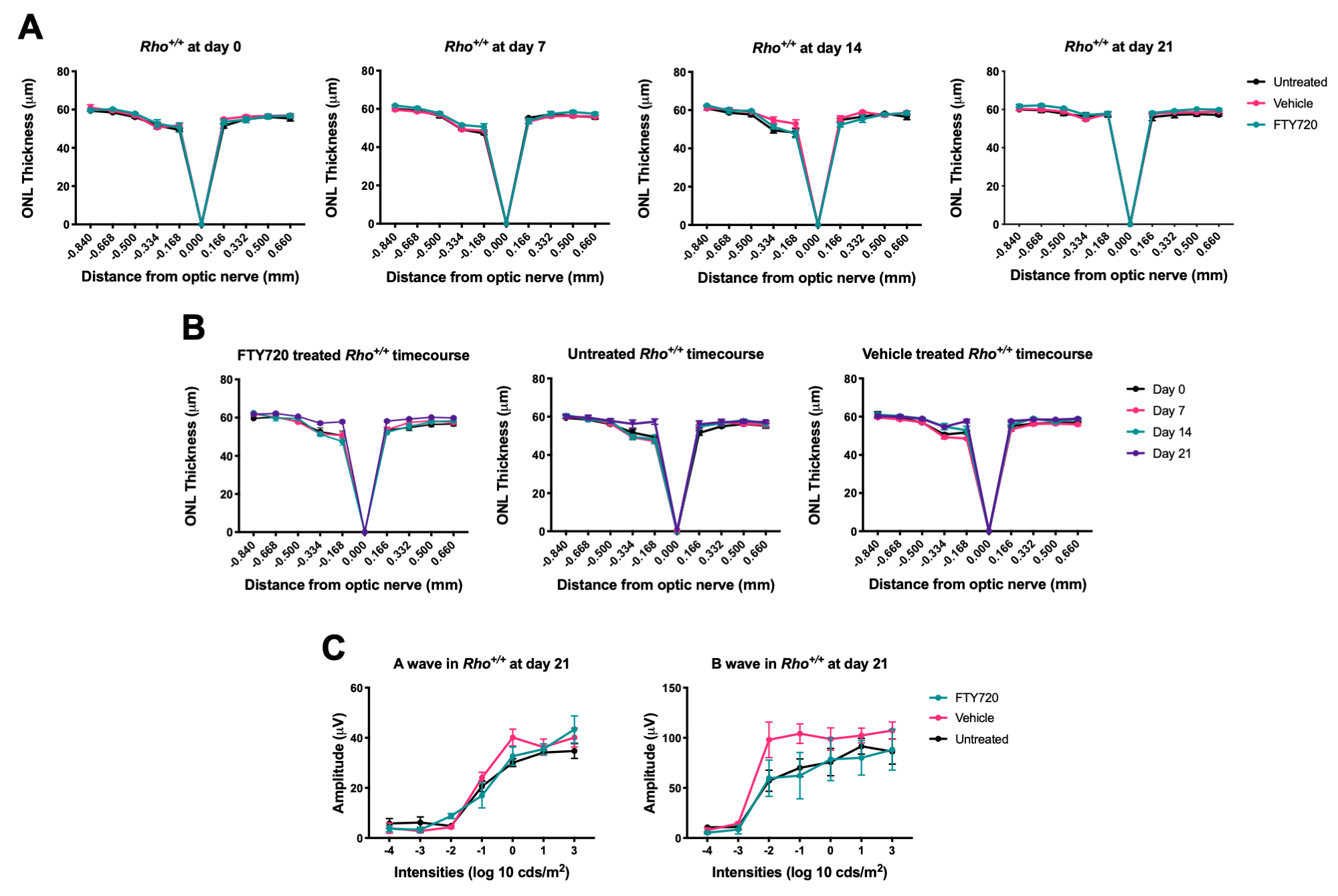
**

**Supplementary Figure 11.** (**A**) ONL thickness of *Rho^+/+^* mouse retina at each round of ERG: day 0, day 7, day 14 and day 21. The measurements were taken by OCT. Dark-adapted mice were intraperitoneally injected with 10 mg/Kg of FTY20 or with vehicle (saline solution) 30 min prior to the ERG. Untreated mice were also analysed. Mean ± SEM. Two-way ANOVA. Tukey’s multiple comparisons test between groups. (**B**) ONL thickness of FTY20 treated, vehicle treated and untreated *Rho^+/+^* KI mice measured over time. Mean ± SEM. Mixed-effect analysis. Dunnett’s multiple comparisons test between groups (**C**) Scotopic A and B wave was also calculated. Mean ± SEM. Two-way ANOVA. Tukey’s multiple comparisons test between groups. Untreated N=3, Vehicle and FTY720 treated N=4.

**
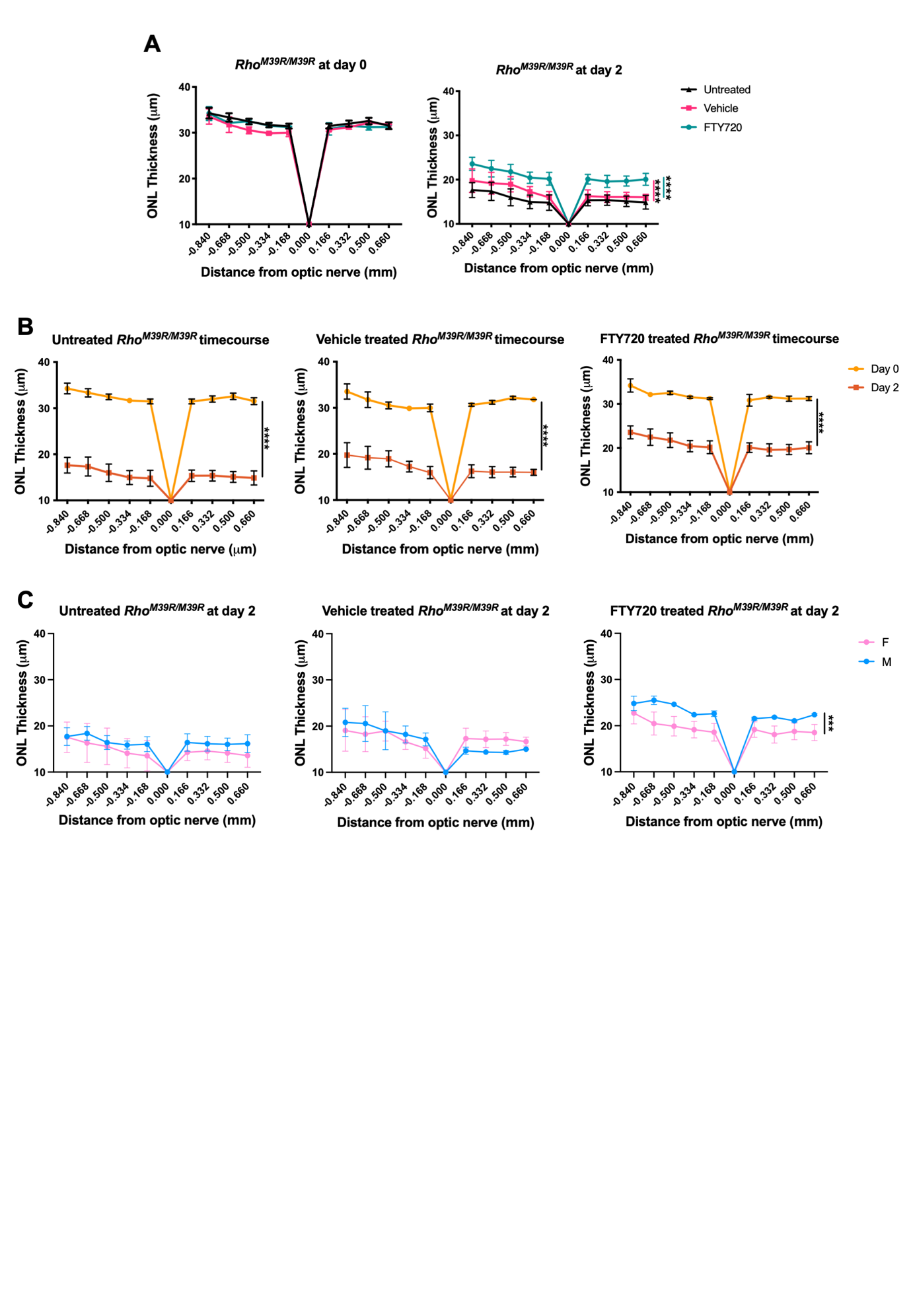
Supplementary Figure 12.** (**A**) ONL thickness of *Rho^M39R/M39R^* KI mouse retina at day 0 and day 2, after a single ERG. Measurements were taken by OCT. Dark-adapted mice were intraperitoneally injected with 10 mg/Kg of FTY20 or with vehicle (saline solution) 30 min prior to the ERG. Mean ± SEM. Two-way ANOVA. Tukey’s multiple comparisons test between groups. (**** p<0.0001). (**B**) ONL thickness of FTY20-treated, vehicle-treated and untreated *Rho^M39R/M39R^* KI mice measured over time. Mean ± SEM. Mixed-effect analysis. (**** p<0.0001). Untreated N=6, Vehicle and FTY720 treated N=5.


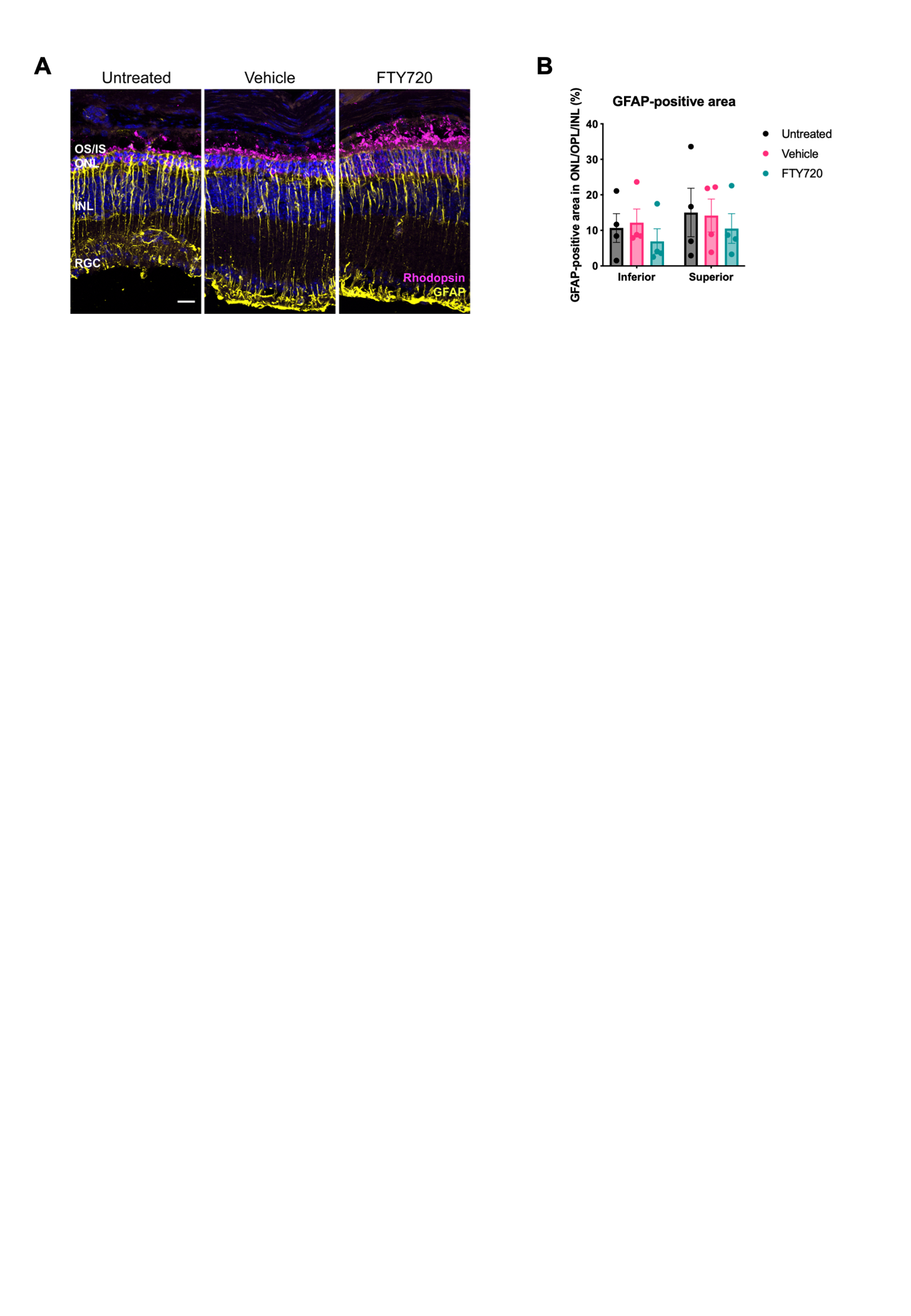


**Supplementary Figure 13.** (**A**) IHC of untreated, vehicle or FTY70 treated *Rho^M39R/M39R^* superior retina after light damage. The cryosections were stained with DAPI, anti-rhodopsin-4D2 (in magenta) and anti-GFAP (in yellow). Scale bar=20μm. (**B**) The % of GFAP-positive area in the ONL, OPL and INL was also measured. Mean ± SEM. Two-way ANOVA. Tukey’s multiple comparisons test between groups. N=4.


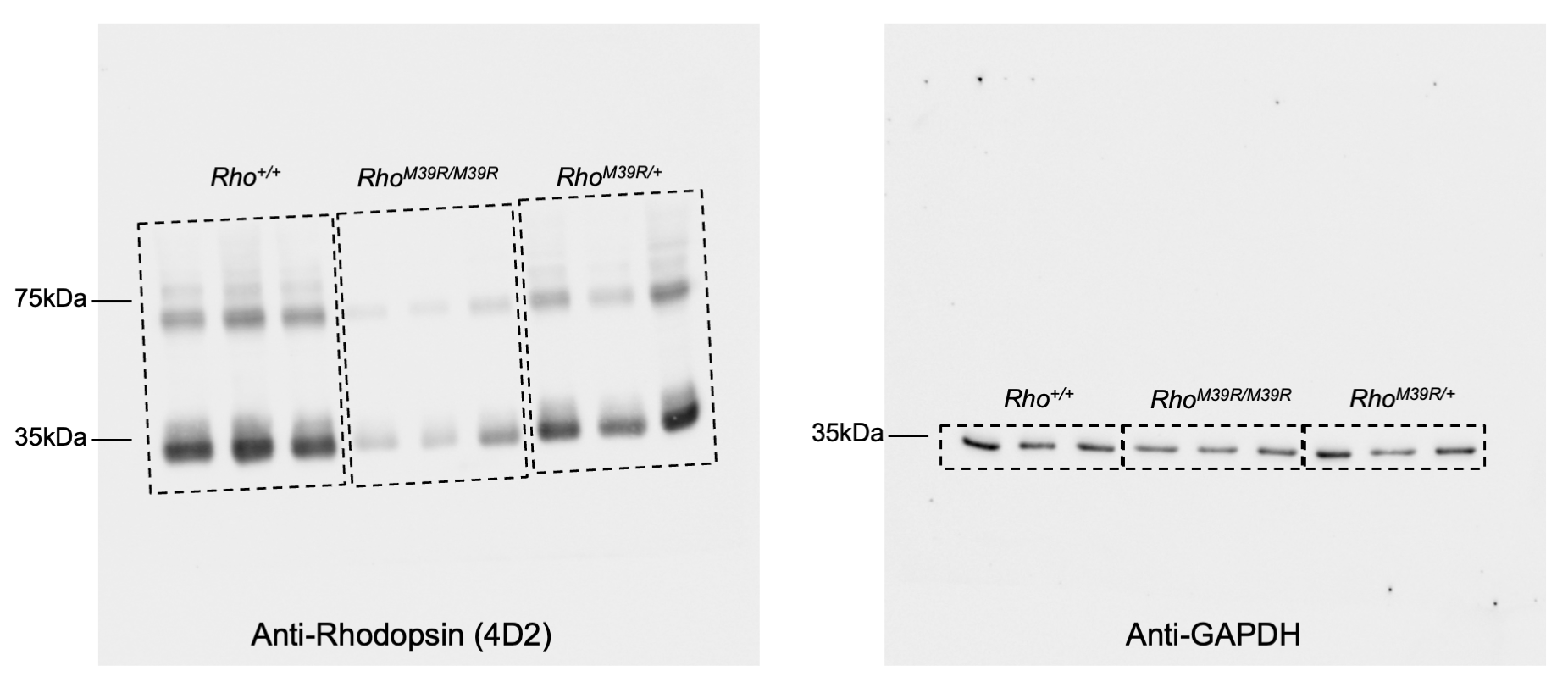


**Supplementary Figure 14.** Uncropped western blot images shown in Supplementary Figure S4.
