# Supplementary figures and images for: Preventing light-induced toxicity in a new mouse model of sector retinitis pigmentosa caused by Rhodopsin M39R variant
Source: Cell Death Discov. 2025 Oct 21;11:477. doi: 10.1038/s41420-025-02769-2 (PMC12540888; doi:10.1038/s41420-025-02769-2)

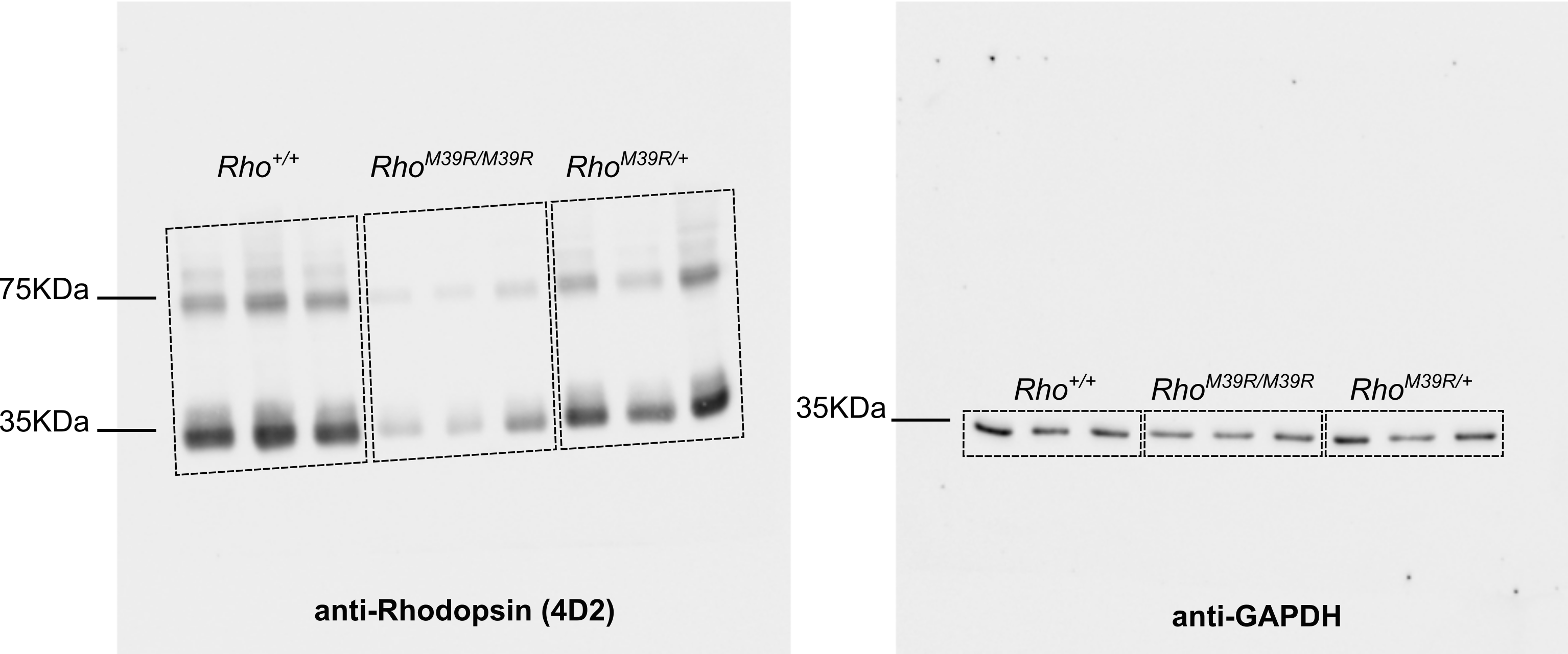

Supplement: Supplementary file 2 — Uncropped Western blot [file 41420_2025_2769_MOESM2_ESM.png]
